# Surgical Quality in Rectal Cancer Management: What Can Be Achieved by a Voluntary Observational Study?

**DOI:** 10.1155/2018/3925062

**Published:** 2018-05-08

**Authors:** Łukasz Dziki, Ronny Otto, Hans Lippert, Paweł Mroczkowski, Olof Jannasch

**Affiliations:** ^1^Department for General and Colorectal Surgery, Medical University Lodz, Pl. Hallera 1, PL-90-647 Łódź, Poland; ^2^Institute for Quality Assurance in Operative Medicine Ltd., Otto-von-Guericke-University, Leipziger Str. 44, D-39120 Magdeburg, Germany; ^3^Department for General and Visceral Surgery, Elisabeth-Hospital, Weinbergstr. 7, D-34117 Kassel, Germany

## Abstract

**Purpose:**

Countries with nationwide quality programmes in colorectal cancer report an improved outcome. In Germany, a self-organized and self-financed observational quality assurance project exists, based on voluntary participation. The object of the present study was to ascertain whether this nationwide project also improves the outcome of colorectal cancer.

**Methods:**

The German Quality Assurance in Colorectal Cancer Project started in 2000 and by 2012 contained 85,000 patients. Inclusion criteria for the study were participation for the entire period of 13 years and treatment of rectal cancer. The following parameters were analysed: (1) patient related: age, gender, ASA classification, T-stage, and N-stage, (2) system related: frequency of preoperative CT and MRI, and (3) outcome related: CRM status, complications, and hospital mortality.

**Results:**

Forty-one of the 345 hospitals treating 11,597 patients fulfilled the inclusion criteria. The median age increased from 67 to 69 years (*p* = 0.002). ASA stages III and IV increased from 32.0% to 37.6% (*p* = 0.005) and from 2.0% to 3.3% (*p* = 0.022), respectively. The use of CT rose from 67.2% to 88.8% (*p* < 0.001) and that of MRI from 5.0% to 35.2% (*p* < 0.001). The proportion of patients suffering from complications decreased from 7.9% to 5.3% (*p* < 0.001) for intraoperative and from 28.0% to 18.6% (*p* < 0.001) for postoperative surgical complications, but general postoperative complications increased from 25.8% to 29.5% (*p* = 0.006). The distribution of histopathological stage, anastomotic leakage, and in-hospital mortality did not change significantly.

**Conclusion:**

Participation in a quality assurance project improves compliance with treatment standards, especially for diagnostic procedures. An improvement of surgical results will require further investment in training.

## 1. Introduction

Surgery is the main therapeutic modality for colorectal cancer, which is the second most common cause of death due to malignancy in Europe [1]. The quality of surgery is not easily measurable, as there are many confounders including factors in the patient, the tumour, the surgeon, and the healthcare system. Since the 1990s, some Nordic countries initiated programmes to audit the results of the treatment of colorectal cancer which when combined with carefully applied training led to improvement in the outcome at a national level [2]. The question remains as to whether this occurred because of changes in the healthcare system or at the hospital level. In countries which have no nationwide audit or focused training in rectal cancer, it is impossible to know whether any change in management is due to the performance of individual hospitals or to the healthcare system. We have analysed the surgical performance of hospitals participating over a 13-year period in a voluntary observational quality assurance programme in an attempt to determine the changes in the quality of practice.

## 2. Method

The project was conducted by the Institute for Quality Assurance in Operative Medicine at the Otto von Guericke University Magdeburg (Germany), which has been previously described [3, 4]. By 2012, the programme included over 85,000 patients. For the purpose of the present analysis, the following inclusion criteria were applied: (1) a diagnosis of rectal cancer defined as a tumour lying below 16 cm from the anal verge and (2) operation in a hospital participating for the entire 13 (2000–2012) years of the study. The following parameters were analysed: (1) patient related: age, gender, American Society of Anesthesiologists (ASA) classification, T-stage, and N-stage, (2) system related: frequency of preoperative CT and MRI, and (3) outcome related: circumferential resection margin- (CRM-) status, anastomotic leakage, intraoperative complications, postoperative surgical and general complications, and hospital mortality. Intraoperative complications were defined as one or more of the following: bladder injury, bleeding necessitating blood transfusion of more than two units, ureteric damage, iatrogenic tumour perforation, splenic injury, intestinal injury, internal genital injury, problem in maintaining the pneumoperitoneum, and any complication of the anastomosis. The postoperative general complications included pulmonary embolism, pleural effusion, atelectasis, pneumonia, urinary tract infection, fever (>38° for more than two days), cardiac, multiple organ failure, thrombosis, and renal failure. The postoperative surgical complications included bleeding necessitating surgery, wound abscess, general sepsis, anastomotic leakage, poor wound healing, wound infection, intra-abdominal or retrorectal abscess, mechanical obstruction necessitating surgery, faecal fistula, peritonitis, paralytic ileus for more than three days, paralytic ileus (not necessitating surgery), wound dehiscence, and stomal complications. All registered data were based on information reported by the participating hospital.

### 2.1. Statistical Analysis

The null hypothesis was that there had been no improvement in outcome over time of the end points of the use of preoperative CT/MRI imaging, the completeness of circumferential margin (CRM) and its positivity rate, the rate of intraoperative and postoperative complications, and mortality. Continuous variables such as age were expressed as median and range. In the first step, we analysed descriptive statistics regarding distribution of the parameters within the cohort of the given year. In the second step, the relationship between two metrical values, the annual results and time (years), was checked using Pearson's correlation coefficient. A *p* value of <0.05 was considered statistically significant. Statistical analysis was performed with IBM® SPSS® Statistics, Version 21.0.0, SPSS Inc. (New York, USA).

This study was approved by the local ethics committee and was undertaken with the understanding and appropriate informed consent of each patient included. Written consent was obtained from each patient.

## 3. Results

Forty-one of the 345 hospitals participating in the study fulfilled the inclusion criteria. Between 2000 and 2012, they treated 11,597 patients with rectal cancer. Over the period, the median age increased from 67 to 70 years (MAD 7000), the proportion of patients of ASA stage I decreased from 10.4% to 5.1%, and the grade of ASA III increased from 32.3% to 39.9%. The use of preoperative CT and MRI increased significantly, although in 2012 MRI was performed in only 37.4% of patients (Table 1).

**Figure 1:**
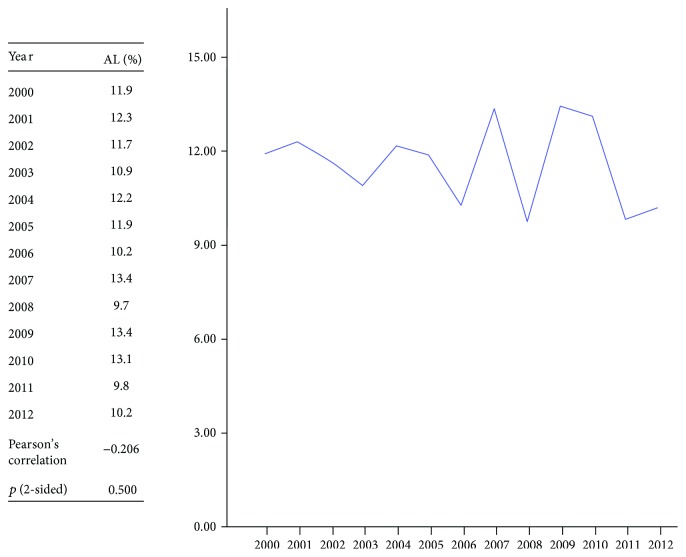
Fluctuation in the rate of anastomotic leakage (AL) between 2000 and 2012.

Histopathological examination of the resected specimen showed a significant fall in the proportion of pT3 and pN2 tumours and a significant increase of stages pT0, pT1, pN0, and pN1. After 13 years, however, more than half of the patients had a locally advanced tumour (stage pT3 or higher) and nearly half were node positive (Table 2). The rate of a positive or unreported CRM did not change significantly. The incidence of an undefined N-stage fell significantly, as did intraoperative and surgical postoperative complications. In contrast, general postoperative complications increased significantly. The hospital mortality did not change significantly, remaining between 2.2% and 4.0% throughout the period (Table 3). The incidence of anastomotic leakage did not change, being between 9.7% and 13.4% (Figure 1).

## 4. Discussion

Various European countries have launched different audit and training projects aimed at quality improvement in colorectal cancer [2, 5]. There are, however, different factors influencing change, including improvement of diagnostic and therapeutic modalities, the maintenance of training and audit, and the evolution of multidisciplinary care.

The median age of 67 to 70 years of the population in the present study corresponded with that reported for other countries including Australia (67.7), Canada (67.8), Denmark (69.3), Norway (70.4), Sweden (70.6), and the United Kingdom (70.4) [6]. Within the system-related parameters, the analysis demonstrated a significantly positive increase in the use of preoperative MRI although this was less than in some other countries. For example, in 2007 24.0% of patients in the present study had an MRI compared with 64.6% in Ireland [7] and the latest achieved level (37.4% in 2012) was still much lower than in other European countries being over 80% in Sweden [8] and Great Britain [9]. This is probably a consequence of there not being a sufficiently strong recommendation for MRI in the German guidelines. Even the 2008 version [10] recommended that MRI should be considered “useful in selected cases.” This has subsequently changed so that in the 2013 version [11] it stated that MRI should be performed “preferably”. The different recommendations for pretreatment staging make international comparison difficult [12].

The quality parameters paint a variable picture. Intraoperative and postoperative surgical complications were reduced, while at the same time general postoperative complications increased significantly. A part of the explanation for this could be the increase of the median age of the patient population and the increase of patients with an advanced ASA grade and tumour stage. This is similar to recent results from Ireland that also report a significant increase in late-stage disease [13]. In a recently published paper describing the Florida Initiative for Quality Cancer Care [14], a spectacular 29.2% reduction of undocumented CRM status between 2006 and 2009 was reported. Even then, the level in 2009 (20.9% undocumented) was still much higher than the 4.3% in the present study. The question remains open regarding the reliability of histopathology, in contrast to Great Britain where there has been a national policy towards standardization of reporting [15]. Unfortunately, Germany does not have a standardized system for the reporting of colorectal surgical specimens, which may make comparability of the results with other databases unreliable.

The incidence of anastomotic leakage did not change over the years and remained within a range of 9.7% to 13.4%. Other international population projects report similar ranges, for example, 10.2% in Japan [16] and 10% in the Netherlands [17]. A Swedish project has shown an overall rate of leakage of 7.3%, but the regional variation is high, ranging from 0% to 33.3% [8]. A Spanish multidisciplinary team training project reported a leakage rate of 8.8%. Leakage may be a good proxy of surgical quality, but we agree that it cannot be regarded as an absolute guide as this will be influenced by the ratio of low anterior resection (with a high risk of leakage) to total anorectal excision with permanent colostomy in which there is no anastomosis. This illustrates the difficulty in comparing such data.

The data in the present study did not show a significant change in the in-hospital mortality. In contrast, the British National Bowel Cancer Audit Project (NBOCAP) reported a fall in the 30-day mortality after major surgical resection of rectal cancer from 4.0% in 2008-9 to 2.9% in 2011-12 [9]. This is exactly the same rate as the hospital mortality observed in the present study for 2012. The Swedish registry for that year reported a lower 30-day mortality rate of 1.3%, and in the Japanese registry for 2011 it was even lower at 0.4%, although the Japanese patients were younger with mean age of 66.2 years [16]. These low rates make the 30-day hospital mortality an insufficiently sensitive parameter for assessing the quality of treatment at the level of a single hospital.

The present study has several limitations. As its primary goal was to determine the change occurring at the level of the care provider (hospital), we decided to choose the longest available period of continuous participation at the cost of reducing the number of hospitals which fulfilled the criteria for entry into the study. On the other hand, the long observation period diminished the bias of staff fluctuations which are inevitable over such a long time. In deciding to concentrate on the longitudinal changes and not the short term impact, it is likely that short term changes will have been obscured by the overall trend. As the use of laparoscopy cannot be treated as a quality parameter, it was not included in the present analysis. Furthermore, we did not consider changes in neoadjuvant treatment, as this would have been given only to the more advanced stages of the disease, and not to the entire population. Unfortunately there are no reports in Germany of patients with rectal cancer who do not have surgery.

Despite the relatively good economic situation of the German health system, there is no general quality audit in rectal cancer; there is also no limitation of hospitals performing this kind of surgery. According to the German Hospital Society (Deutsche Krankenhausgesellschaft, (http://dkg.promato.de/de/suche/search/diseaseAndOperation/operation.html), there are 1087 hospitals performing low anterior resection (procedure code: 5-484.5, requested on August 16th, 2015). With all the limitations and deficiencies of the German Quality Assurance in Colorectal Cancer Project, it is the only available clinical quality assessment in Germany. As 42% of German hospitals ended the year 2013 in financial deficit and 38.7% expected a further deterioration in the future [18], there is no reason to believe that hospitals will be in a position to make the additional effort to establish audit programmes, especially owing to their cost and the shortage of doctors. Some years ago the German Cancer Society initiated a project of “certified bowel cancer centers” [19]. The main part of this concept concerned procedural quality: presentation in the multidisciplinary team meeting, psychooncological and social counselling, and so on. The clinical parameters are given as defined “targets”, which should be reached in order to maintain the certified status. We have previously compared the performance of certified and noncertified centers participating in our project [20], seeing no significant difference in outcome. The influence of the “certified centers” project on quality of care is still a controversial matter for discussion in Germany [21].

Despite the known disparities in outcome [22], there is no compete international consensus regarding the treatment strategy of colorectal cancer [23]. Much effort has been made to use administrative data as a proxy for quality [24–26], but their value is limited [27–30]. Clinically-based quality measures are present in some countries [5], but most patients are treated outside any system of quality control. Participation in a quality assessment programme facilitates the introduction of standards for new treatments as they become available, but the improvement of the results of surgery requires formal training and audit. A comparison between standardized pretherapeutic MRI, as a prediction of the locoregional histopathology with a standardized postoperative histopathology report by the pathologist, combined with quality of life monitoring is one of the goals of the assurance of surgical quality.

## Figures and Tables

**Table 1:** Changes between 2000 and 2012 of patient-related factors including age, gender, ASA stage, and pretreatment imaging.

Year	Age/MAD (years)	Male (%)	Female (%)	ASA I (%)	ASA II (%)	ASA III (%)	ASA IV (%)	CT (%)	MRI (%)
2000	67.00 7.0000	58.7	41.3	10.4	55.4	32.3	2.0	66.5	4.8
2001	67.00 7.0000	57.5	42.5	11.6	51.2	35.4	1.8	73.0	7.8
2002	66.00 7.0000	59.3	40.7	11.2	52.6	34.2	2.0	73.5	7.2
2003	67.00 7.0000	56.4	43.6	9.5	54.1	33.9	2.4	74.6	7.8
2004	68.00 7.0000	59.3	40.7	8.6	51.8	36.5	3.1	75.7	11.8
2005	68.00 7.0000	59.6	40.4	7.2	51.0	39.7	2.1	82.3	15.2
2006	68.00 6.0000	59.6	40.4	6.8	52.2	39.6	1.4	82.4	17.4
2007	69.00 7.0000	61.9	38.1	6.5	49.5	42.0	2.0	80.4	24.0
2008	68.00 8.0000	60.8	39.2	7.5	48.7	41.5	2.3	82.3	26.7
2009	69.00 8.0000	62.4	37.6	5.7	52.1	39.6	2.6	84.3	30.6
2010	70.00 7.0000	61.8	38.2	6.2	47.3	42.9	3.7	84.3	36.2
2011	69.00 8.0000	61.7	38.3	7.2	51.4	38.2	3.3	88.4	37.1
2012	70.00 7.0000	62.7	37.3	5.1	51.6	39.9	3.5	85.8	37.4
Pearson's correlation coefficient	***0.899↑***	***0.862↑***	***−0.862↓***	***−0.892↓***	***−0.575↓***	***0.783↑***	***0.673↑***	***0.939↑***	***0.982↑***
*p* (2-sided)	***<0.001***	***<0.001***	***<0.001***	***<0.001***	***0.040***	***0.002***	***0.012***	***<0.001***	***<0.001***

MAD: median absolute deviation.

**Table 2:** Changes between 2000 and 2012 of histopathologic tumour stage.

Year	pT0 (%)	pT1 (%)	pT2 (%)	pT3 (%)	pT4 (%)	pN0 (%)	pN1 (%)	pN2 (%)
2000	1.6	9.0	25.3	51.7	6.5	50.3	17.7	21.7
2001	2.0	10.6	26.7	50.2	6.1	53.3	19.7	18.6
2002	2.1	8.4	29.2	48.5	6.6	52.1	17.6	20.5
2003	2.0	11.6	26.0	47.2	6.1	50.4	19.2	19.8
2004	2.9	10.9	26.1	47.1	7.1	52.1	20.8	16.6
2005	2.1	10.6	28.5	48.6	5.6	54.0	20.8	18.4
2006	1.3	11.5	29.0	45.9	6.3	54.9	20.8	16.8
2007	2.5	14.4	27.2	47.0	5.1	59.9	17.5	17.9
2008	4.2	12.2	24.1	44.9	10.2	53.8	21.2	18.2
2009	3.2	9.6	26.1	48.7	10.2	54.5	21.1	19.2
2010	2.9	14.0	23.9	45.9	10.5	54.4	20.4	19.2
2011	5.1	11.3	25.9	48.4	6.5	54.7	21.1	18.0
2012	4.7	12.3	29.1	46.8	5.6	58.5	20.7	14.9
Pearson's correlation coefficient	***0.787↑***	***0.563↑***	**−0.089**	***−0.580↓***	**0.343**	***0.672↑***	***0.593↑***	***−0.591↓***
*p* (2-sided)	***<0.001***	***0.045***	**0.772**	***0.038***	**0.251**	***0.012***	***0.033***	***0.033***

**Table 3:** Changes between 2000 and 2012 of mortality and morbidity and histopathological parameters.

Year	CRM n/a (%)	CRM (+) (%)	pTx (%)	pNx (%)	IntraOP (%)	Surgical (%)	General (%)	Lethality (%)
2000	6.5	6.3	0.6	1.1	8.4	27.4	26.1	2.2
2001	4.0	3.9	0.7	0.9	9.3	28.8	25.3	2.2
2002	3.2	3.7	1.0	1.4	8.9	27.5	28.0	2.9
2003	3.4	3.0	1.5	1.8	6.0	23.2	23.7	3.8
2004	3.4	3.1	1.8	1.2	7.0	28.2	25.8	2.8
2005	1.2	3.4	0.1	0.2	6.9	23.5	28.8	2.8
2006	1.7	4.0	2.1	1.5	6.4	24.0	26.9	3.0
2007	6.5	4.6	1.5	0.3	6.6	20.4	28.1	2.2
2008	3.0	4.0	1.6	0.4	4.4	18.5	26.6	3.1
2009	4.3	2.9	1.6	0.3	5.2	19.9	27.1	2.6
2010	3.0	3.4	1.3	0.0	4.8	17.9	30.0	4.1
2011	6.3	3.5	1.3	0.4	5.2	18.9	29.6	3.4
2012	7.2	2.3	0.6	1.1	3.6	20.0	30.0	2.9
Pearson's correlation coefficient	**0.247**	**−0.543**	**0.207**	**−0.535**	***−0.899↓***	***0.885↓***	***0.707↑***	**0.419**
*p* (2-sided)	**0.415**	**0.055**	**0.497**	**0.060**	***<0.001***	***<0.001***	***0.007***	**0.154**

CRM n/a: CRM not defined; CRM (+): CRM positive; pTx: pT not defined; pNx: pN not defined; IntraOP: intraoperative complications; surgical: postoperative surgical complications; general: postoperative general complications; lethality: hospital mortality.

## Data Availability

The data are part of a quality assurance project, as described in the article.
